# Analysis of codon usage bias of lumpy skin disease virus causing livestock infection

**DOI:** 10.3389/fvets.2022.1071097

**Published:** 2022-12-05

**Authors:** Siddiq Ur Rahman, Hassan Ur Rehman, Inayat Ur Rahman, Abdur Rauf, Abdulrahman Alshammari, Metab Alharbi, Noor ul Haq, Hafiz Ansar Rasul Suleria, Sayed Haidar Abbas Raza

**Affiliations:** ^1^Department of Computer Science and Bioinformatics, Khushal Khan Khattak University, Karak, Pakistan; ^2^Department of Botany, Khushal Khan Khattak University, Karak, Pakistan; ^3^Department of Chemistry, University of Swabi, Swabi, Pakistan; ^4^Department of Pharmacology and Toxicology, College of Pharmacy, King Saud University, Riyadh, Saudi Arabia; ^5^Faculty of Veterinary and Agricultural Sciences, School of Agriculture and Food, The University of Melbourne, Melbourne, VIC, Australia; ^6^College of Animal Science and Technology, Northwest A&F University, Xianyang, China; ^7^Safety of Livestock and Poultry Products, College of Food Science, South China Agricultural University, Guangzhou, China

**Keywords:** lumpy skin disease virus, codon usage bias (CUB), effective number of codons (ENC), mutation pressure, natural selection

## Abstract

Lumpy skin disease virus (LSDV) causes lumpy skin disease (LSD) in livestock, which is a double-stranded DNA virus that belongs to the genus *Capripoxvirus* of the family *Poxviridae*. LSDV is an important poxvirus that has spread out far and wide to become distributed worldwide. It poses serious health risks to the host and causes considerable negative socioeconomic impact on farmers financially and on cattle by causing ruminant-related diseases. Previous studies explained the population structure of the LSDV within the evolutionary time scale and adaptive evolution. However, it is still unknown and remains enigmatic as to how synonymous codons are used by the LSDV. Here, we used 53 LSDV strains and applied the codon usage bias (CUB) analysis to them. Both the base content and the relative synonymous codon usage (RSCU) analysis revealed that the AT-ended codons were more frequently used in the genome of LSDV. Further low codon usage bias was calculated from the effective number of codons (ENC) value. The neutrality plot analysis suggested that the dominant factor of natural selection played a role in the structuring of CUB in LSDV. Additionally, the results from a comparative analysis suggested that the LSDV has adapted host-specific codon usage patterns to sustain successful replication and transmission chains within hosts (*Bos taurus* and *Homo sapiens*). Both natural selection and mutational pressure have an impact on the codon usage patterns of the protein-coding genes in LSDV. This study is important because it has characterized the codon usage pattern in the LSDV genomes and has provided the necessary data for a basic evolutionary study on them.

## Introduction

Lumpy skin disease virus (LSDV) causes lumpy skin disease (LSD), which is a viral disease affecting the ruminants, while the virus itself belongs to the genus *Capripoxvirus* of the family *Poxviridae* (1). This disease assumes economic significance due to the associated financial destruction to farmers it brings along and has a genome that consists of the double-stranded DNA (2). It is genetically associated with the sheeppox and goatpox virus family *Poxviridae*. The lumpy skin disease causing LSDV is transmitted to animals through blood-sucking arthropods while infecting a human being through direct transmission without the need for a vector (3, 4).

Lumpy skin disease is a viral disease predominantly attacking the bovine population, which presents a plethora of specific clinical signs ranging from subclinical infection to death (4). The main clinical signs and symptoms predictive of this disease are fever, mouth lesions, pharynx, nodules' appearance in the skin, skin edema, and enlargement of the superficial lymph nodes (3–5). The affected livestock instantly start losing weight and produce less milk. However, in the severest of severe cases, the livestock even die from an LSDV infection (1). LSDV gets distributed so widely that it has pervaded almost the entire globe and consequently thereof has set off a wave of serious economic problems for the affected countries worldwide. LSDV was first identified and reported in cattle in an outbreak in Zambia in 1929 (5), and the genome of the LSDV appeared to remain stable there for many years. Only minor genetic changes were noticed between the LSDV field isolates that were recovered over many years in Africa. However, the first outbreaks of this deadly disease surfaced in Egypt in 1988, which further spread to the Middle East in 2012 and later to Europe in 2015. The incidence of the disease has been further identified in a country as far as Russia, prevailing there from 2017 to 2019 (6). Recently, the disease has started wrecking havoc in Asia too, especially in Pakistan, where the prevalence of LSD has been steadily increasing; from 2020 until now, five million dairy farmers have suffered huge losses in the aftermath of LSD's attack on crops (7). Previous studies suggested that LSDV transmission spreads across the countries when the infected cattle are moved from one place to another or by vectors in animal trucks (6). Therefore, it is essential to use genomic analysis based on the knowledge of the distributing LSDV strains while considering the increasing diversity of LSDV in recent years.

Codon usage bias (CUB) refers to a phenomenon in which synonymous codons are not used with an equal frequency during gene translation. CUB is a common phenomenon that is observed in numerous species, including prokaryotes, eukaryotes, and viruses (8, 9). We noticed that a variety of factors affect how codons are used by different organisms. The primary explanations put forth for this variance in codon usage among the genes in these species are attributed to be due to weak natural selection and mutational pressure (10). Extensive research into codon usage patterns across the entire genome is imperative for understanding the fundamental characteristics of a genome's molecular organization. Furthermore, CUB analysis took into account numerous other crucially applied aspects, including heterologous gene expression (11), identifying species origins (12), predicting gene expression levels (13, 14), and predicting gene functions (15). However, the majority of the numerous reports on CUB concentrated on numerous microorganisms and viruses, including *Betacoronavirus* (16) and *Henipavirus* (17). For instance, we need to take note of the fact that the most preferred codons in Porcine astrovirus end in A or U (18). On the other hand, genome-wide studies on LSDV are limited.

The pattern of usage of synonymous codons by LSDV is fraught with uncertainty. In this study, we used a multivariate statistical analysis to examine the codon usage patterns of LSDV using the complete coding data. The analysis of the codon usage patterns of LSDV makes it possible for us to understand the underlying mechanism behind the biased usage of synonymous codons and to select suitable host expression systems for an optimal expression of the target genes.

## Materials and methods

### Sequences

A total of 53 complete coding sequences (CDS) of the lumpy skin disease virus (LSDV) were retrieved from the National Center for Biotechnology Information (NCBI) GenBank database (https://ncbi.nlm.nih.gov/nuccore/?term=Lumpy+Skin+Disease+Virus). The number of nucleotides in the coding sequence was an exact multiple of 3 (19). Complete information about the overall 53 LSDV strains associated with Asian, African, and European countries is listed in Supplementary Table 1.

### Analysis of the nucleotide composition

Here, we employed the CodonW software to determine the total base composition (G, C, A, and T%) and the nucleotide contents at the 3rd codon position (C3, T3, G3, and A3%) for all synonymous codons in LSDV. The GC% contents of all three codon positions (GC1, GC2, and GC3%) were measured. Additionally, the average frequency of G/C at GC12 positions and the overall GC/AT compositions were also determined. Furthermore, only 59 synonymous codons encoding 18 amino acids were considered for the present study, not including the 1st ATG codon, the codon (TGG) encoding tryptophan, and the three end codons (TAG, TAA, and TGA), respectively (12).

### Analysis of the relative synonymous codon usage

The RSCU values indicate the observed codon occurrence to its random occurrence, suggesting that all the identical codons of the LSDV are equal in usage. There are frequently occurring codons that have an RSCU value >1 in the CDS, and less frequently occurring codons that have an RSCU value <1 in the CDS (20). Higher CUB or more frequently used codons were determined through high RSCU. In the coding sequence, the overrepresented codon represents the codon RSCU value >1.6, and the underrepresented codon represents the codon RSCU value <0.6 (21). The RSCU value was determined for each codon by using the following formula (22):


$$\begin{array}{l} RSCU=\frac{X_{ij}}{\sum_{j=1}^{n_{i}}X_{ij}}n_{i} \end{array}$$

Here, n_i_ is the number of codons for the ith amino acid and X_ij_ denotes the frequency of the jth codon for the ith amino acid.

### Indices of codon usage

To determine the proper measurement of a codon bias, ENC (effective number of codons) value, which measures the total usage of the codon in a certain gene, was calculated (30, 31). It clarifies the ratio of codon variation in a gene from the total even usage of codons, which are synonymous. The ENC value varies from 20 (where one amino acid encodes one codon only) to 61 (where each amino acid is used randomly for all codons). The ENC value of <35 implies significant CUB (23, 25). To determine the impact of GC3s composition on the codon usage, a plot is drawn out between ENC and GC3s (23). For each GC3, the expected ENC values were computed by using the following formula:


$$\begin{array}{l} \text{ENC}=2+\frac{9}{F_{2}}+\frac{1}{F_{3}}+\frac{5}{F_{4}}+\frac{3}{F_{6}}\\ F=\;\frac{n\sum_{i=1}^{k}p_{i}^{2}-1}{n-1}\;n>1\;p_{i}=\;\frac{n_{i}}{n}, \end{array}$$

where *n* is the total number of observations of the codons for that amino acid and *ni* is the total number of events of the *i*th codon for that amino acid.


$$\begin{array}{l} ENC=2+s+\frac{29}{\left(s^{2}+{\left(1-s\right)}^{2}\right)} \end{array}$$

wherever “s” is the GC3s content of each codon.

### Analysis of the neutrality plot

The obtained GC3 and GC12 values were plotted to determine and compare the extent of the factors that influence the preference of codon usage in the graph, each point signifying a discrete gene. The line of regression slope between the GC3 and the GC12 indicates that the mutational pressure is the major factor affecting CUB, i.e., for values coming close to 1, although if the value comes close to 0, it indicates that the selection pressure has been the main factor in defining CUB (26, 27).

### Analysis of codon adaptation index

The codon adaptation index (CAI) is applied to calculate the gene expression level depending on its codon-based sequence. The value of the CAI varies from 0 to 1; a value near 1 indicates higher levels of codon usage bias (CUB) (28). The CAI was determined through an online tool used for the CAI calculation (http://genomes.urv.es/CAIcal) (29), where the *Bos taurus* and *Homo sapiens* genomes were used as reference sources. Furthermore, the e-CAI (expected CAI) was analyzed using the online tool “http://genomes.urv.es/CAIcal/.” The RSCU values for the *Bos taurus* and *Homo sapiens* genomes were retrieved from the codon usage database.

### Correspondence analysis

The correspondence analysis (COA) is a multidimensional critical method that is used to resolve important developments in the codon usage patterns of CDS through codon RSCU values (12, 30, 31). To create the COA plot, the RSCU values of 59 codons were considered. To study the tendencies in the deviation of the use of codon, relative inertia was used to hold a specific position in the graph.

### Phylogenetic analysis of LSDV

The phylogenetic tree was constructed using the maximum-likelihood (ML) method in Clustal × 2 (http://www.clustal.org/clustal2/). The phylogenetic tree was designed using the online tool, the Interactive Tree of Life (iTOL) version 3 (http://itol.embl.de/). A total of 53 strains were used in this study.

### Correlation analysis

To illustrate the relationships between the nucleotide content and codon usage patterns, an LSDV correlation analysis was performed. These analyses were conducted by using Spearman's rank correlation method (32). All processes were executed using the R corrplot package. For codon usage index analysis, CodonW (1.4.4) software was applied (33, 34) to simplify the Multivariate analysis (correspondence analysis) of codon and amino acid usage.

### Similarity index analysis

The similarity index (SiD), which was used to measure how the overall codon usage pattern of the host affects the overall codon usage of the virus, was determined as follows:


$$\begin{array}{l} \text{R}\left(\text{A},\text{B}\right)=\;\frac{\sum_{i=1}^{59}a_{i}\times b_{i}}{\sqrt{\sum_{i=1}^{59}a_{i}^{2}\times \sum_{i=1}^{59}b_{i}^{2}}} \end{array}$$

$$\begin{array}{l} D\left(A,B\right)=\frac{1-R\left(A,B\right)}{2}, \end{array}$$

where the R(A, B) denotes the degree of similarity between the overall codon usage patterns of the host and LSDV, which is defined as the cosine value of the angle between A and B. Among the 59 synonymous codons in the LSDV, a_i_ is defined as the RSCU value for a particular codon. The RSCU value for the host's identical codon is known as b_i_. The value of D(A,B) ranges from 0 to 1.0 and shows the potential impact of the host's total codon usage on that of LSDV (35).

## Results and discussion

### Basic compositional analysis in lumpy skin disease virus coding sequences

**Figure 1:**
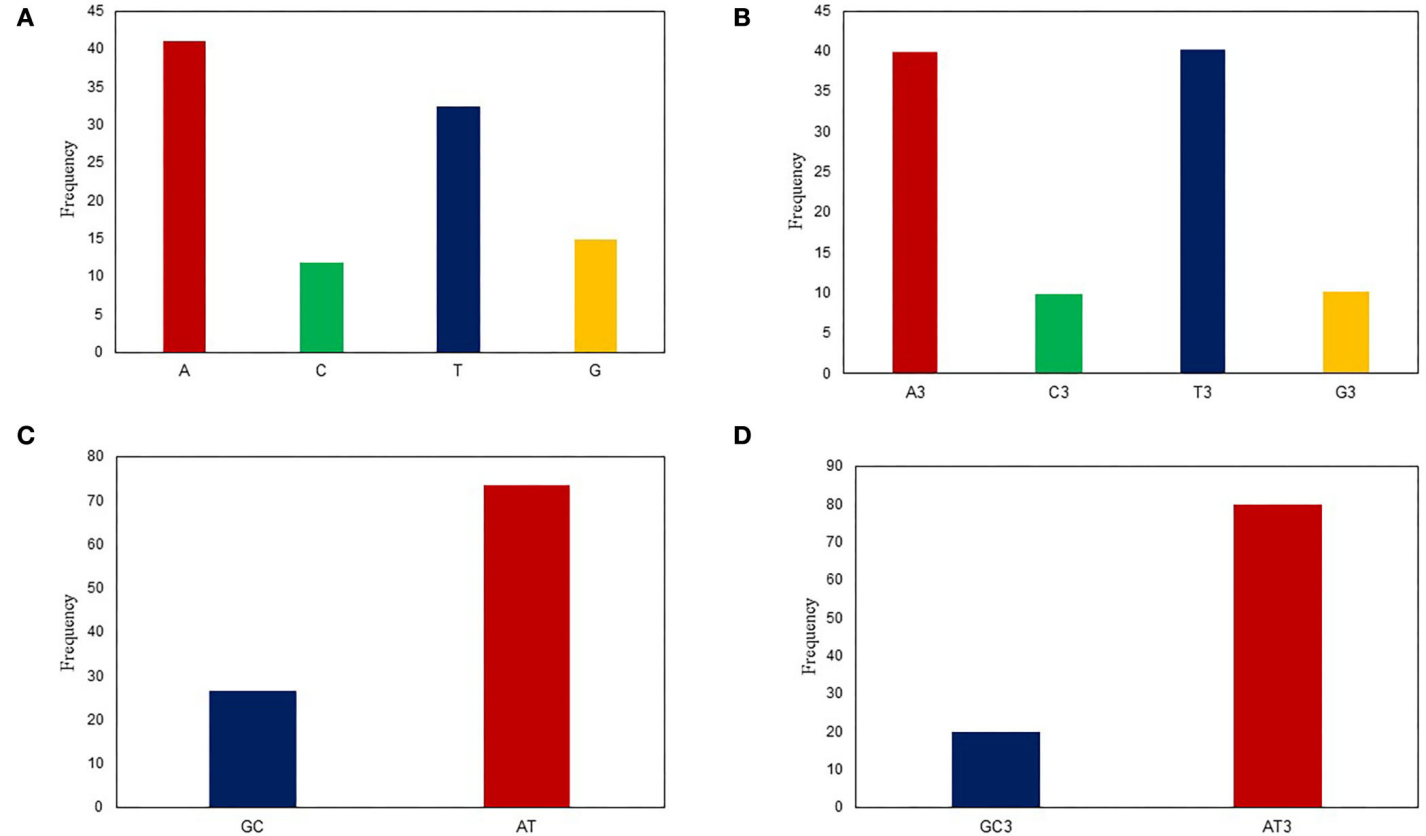
Nucleotide composition analysis: **(A)** The average values of the A, T, G, and C nucleotide composition of the entire viral genome. **(B)** The average values of the nucleotide composition at the 3rd codon position, indicating A/T richness followed G/C richness. **(C)** The mean frequency of GC and AT composition. **(D)** The mean frequency of GC and AT composition at the 3rd codon position, indicating that AT3 is more common than GC3.

Codon usage bias (CUB) can be considerably predisposed by the general base composition of genomes. The nucleotide contents of 53 LSDV strains were studied and are presented in Table 1. Here, our results showed that the mean A (41.0%) and T (32.37%) were maximum, tailed by G (14.8%) and C (11.73%), across all genomes (Figure 1A). The mean A3 (39.83%) and T3 (40.2%) occurred at a maximum level higher than the G3 (10.1%) and C3 (9.81%) (Figure 1B). The total AT and GC compositions were found to be 73.42 and 26.57%, respectively, suggesting that LSDV strains have strong AT (Figure 1C). This finding is similar to the previous research on Porcine astrovirus (18), Hantaan virus (36), and Bluetongue virus (12) which were enriched with A and T. However, the biological significance of this condition still needs to be clarified, and therefore, it is essential to explore the causes for increased AT contents and decreased GC contents in the virus genomes (37).

**Table 1:** Nucleotide compositional analysis of lumpy skin disease virus (LSDV) coding sequences (%).

**Sequences**	**A**	**C**	**T**	**G**	**GC**	**AT**	**GC1**	**GC2**	**A3**	**C3**	**T3**	**G3**	**GC3**	**AT3**	**ENC**
MN864146.1	45.39	12.62	28.96	13.03	25.65	74.35	28.64	24.03	44.42	10.92	31.31	13.35	24.27	75.73	43.6
MW251476.1	46.53	13.04	26.4	14.03	27.06	72.94	33.17	28.71	45.54	11.39	35.15	7.92	19.31	80.69	37.9
MW251475.1	32.81	13.09	37.96	16.14	29.23	70.77	30.1	34.82	30.63	9.16	46.6	13.61	22.77	77.23	44.1
FJ869377.1	33.07	12.61	38.1	16.23	28.84	71.16	29.89	34.39	31.22	8.47	46.56	13.76	22.22	77.78	43.5
MZ934387.1	38.91	9.91	36.02	15.17	25.08	74.92	31.27	25.7	38.7	7.74	43.03	10.53	18.27	81.73	36.8
MZ934386.1	46.53	13.04	26.4	14.03	27.06	72.94	33.17	28.71	45.54	11.39	35.15	7.92	19.31	80.69	37.9
MZ934385.1	32.81	13.09	37.96	16.14	29.23	70.77	30.1	34.82	30.63	9.16	46.6	13.61	22.77	77.23	44.1
MN422450.1	39.01	10.11	35.6	15.27	25.39	74.61	31.27	26.01	39.01	8.05	42.11	10.84	18.89	81.11	37.1
MN422448.1	38.39	10.53	35.6	15.48	26.01	73.99	32.51	26.63	38.7	8.36	42.41	10.53	18.89	81.11	38.1
MN422451.1	39.01	10.11	35.71	15.17	25.28	74.72	31.27	26.01	39.01	8.05	42.41	10.53	18.58	81.42	36.3
MN598006.1	32.72	13.18	37.96	16.14	29.32	70.68	30.1	34.82	30.37	9.42	46.6	13.61	23.04	76.96	44.2
MN598005.1	38.91	9.91	36.02	15.17	25.08	74.92	31.27	25.7	38.7	7.74	43.03	10.53	18.27	81.73	36.8
MK302113.1	46.37	12.87	26.57	14.19	27.06	72.94	33.17	28.71	45.05	10.89	35.64	8.42	19.31	80.69	38.4
MK302103.1	46.37	12.87	26.57	14.19	27.06	72.94	33.17	28.71	45.05	10.89	35.64	8.42	19.31	80.69	38.4
MK302111.1	46.37	12.87	26.57	14.19	27.06	72.94	33.17	28.71	45.05	10.89	35.64	8.42	19.31	80.69	38.4
OM674465.1	46.37	13.04	26.4	14.19	27.23	72.77	33.66	28.71	45.05	10.89	35.64	8.42	19.31	80.69	38.2
OM674464.1	46.37	13.04	26.4	14.19	27.23	72.77	33.66	28.71	45.05	10.89	35.64	8.42	19.31	80.69	38.2
OM674463.1	46.37	13.04	26.4	14.19	27.23	72.77	33.66	28.71	45.05	10.89	35.64	8.42	19.31	80.69	38.2
OM674462.1	46.37	13.04	26.4	14.19	27.23	72.77	33.66	28.71	45.05	10.89	35.64	8.42	19.31	80.69	38.2
OM674461.1	46.37	13.04	26.4	14.19	27.23	72.77	33.66	28.71	45.05	10.89	35.64	8.42	19.31	80.69	38.2
OM674460.1	46.37	13.04	26.4	14.19	27.23	72.77	33.66	28.71	45.05	10.89	35.64	8.42	19.31	80.69	38.2
OL741677.1	46.37	13.04	26.4	14.19	27.23	72.77	33.17	28.71	45.05	11.39	35.15	8.42	19.8	80.2	38.6
OL741676.1	46.37	13.04	26.4	14.19	27.23	72.77	33.17	28.71	45.05	11.39	35.15	8.42	19.8	80.2	38.6
OL741675.1	46.37	13.04	26.4	14.19	27.23	72.77	33.17	28.71	45.05	11.39	35.15	8.42	19.8	80.2	38.6
OL741674.1	46.37	13.04	26.4	14.19	27.23	72.77	33.17	28.71	45.05	11.39	35.15	8.42	19.8	80.2	38.6
OL741673.1	46.37	13.04	26.4	14.19	27.23	72.77	33.17	28.71	45.05	11.39	35.15	8.42	19.8	80.2	38.6
OL741672.1	39.15	8.81	36.79	15.25	24.06	75.94	34.43	20.75	34.43	8.02	48.58	8.96	16.98	83.02	41.5
OL741671.1	39.15	8.81	36.79	15.25	24.06	75.94	34.43	20.75	34.43	8.02	48.58	8.96	16.98	83.02	41.5
OL741670.1	39.15	8.81	36.79	15.25	24.06	75.94	34.43	20.75	34.43	8.02	48.58	8.96	16.98	83.02	41.5
OL741669.1	39.15	8.81	36.79	15.25	24.06	75.94	34.43	20.75	34.43	8.02	48.58	8.96	16.98	83.02	41.5
OL741668.1	39.15	8.81	36.79	15.25	24.06	75.94	34.43	20.75	34.43	8.02	48.58	8.96	16.98	83.02	41.5
MK302107.1	46.37	12.87	26.57	14.19	27.06	72.94	33.17	28.71	45.05	10.89	35.64	8.42	19.31	80.69	38.4
MH271111.1	43.08	9.75	31.97	15.2	24.95	75.05	30.41	22.81	44.44	9.94	33.92	11.7	21.64	78.36	37.5
MH271109.1	43.27	9.75	31.97	15.01	24.76	75.24	29.82	22.81	44.44	9.94	33.92	11.7	21.64	78.36	37.6
MG757480.1	46.37	12.87	26.57	14.19	27.06	72.94	33.17	28.71	45.05	10.89	35.64	8.42	19.31	80.69	38.4
KY595106.1	33.07	12.61	38.1	16.23	28.84	71.16	29.89	34.39	31.22	8.47	46.56	13.76	22.22	77.78	43.5
KJ818290.1	46.37	13.04	26.4	14.19	27.23	72.77	33.66	28.71	45.05	10.89	35.64	8.42	19.31	80.69	38.2
MW748479.1	33.07	12.61	38.1	16.23	28.84	71.16	29.89	34.39	31.22	8.47	46.56	13.76	22.22	77.78	43.5
MW344043.1	33.07	12.7	38.1	16.14	28.84	71.16	29.89	34.39	31.22	8.47	46.56	13.76	22.22	77.78	43.5
MH639094.1	41.62	10.12	34.55	13.7	23.82	76.18	26.96	25.13	39.01	9.95	41.62	9.42	19.37	80.63	41.2
FJ869370.1	33.07	12.7	38.01	16.23	28.92	71.08	29.89	34.66	31.22	8.47	46.56	13.76	22.22	77.78	43.7
OL689586.1	46.37	13.04	26.4	14.19	27.23	72.77	33.66	28.71	45.05	10.89	35.64	8.42	19.31	80.69	38.2
OL689584.1	46.37	13.04	26.4	14.19	27.23	72.77	33.66	28.71	45.05	10.89	35.64	8.42	19.31	80.69	38.2
MW815879.1	38.91	10.11	35.71	15.27	25.39	74.61	31.58	26.01	39.01	8.05	42.41	10.53	18.58	81.42	36.3
MW452621.1	39.01	10.22	35.6	15.17	25.39	74.61	31.27	26.01	39.01	8.36	42.11	10.53	18.89	81.11	36.4
MW452620.1	39.01	10.22	35.6	15.17	25.39	74.61	31.27	26.01	39.01	8.36	42.11	10.53	18.89	81.11	36.4
MW452619.1	39.01	10.22	35.6	15.17	25.39	74.61	31.27	26.01	39.01	8.36	42.11	10.53	18.89	81.11	36.4
MW452615.1	39.01	10.22	35.6	15.17	25.39	74.61	31.27	26.01	39.01	8.36	42.11	10.53	18.89	81.11	36.4
MW452618.1	39.01	10.22	35.6	15.17	25.39	74.61	31.27	26.01	39.01	8.36	42.11	10.53	18.89	81.11	36.4
OL692421.1	33.45	17.01	35.27	14.27	31.28	68.72	31.85	30.82	34.59	21.58	34.25	9.59	31.16	68.84	48.2
LC648887.1	38.91	10.01	36.02	15.07	25.08	74.92	31.27	25.7	38.7	7.74	43.03	10.53	18.27	81.73	36.6
LC663765.1	38.91	9.91	36.02	15.17	25.08	74.92	31.27	25.7	38.7	7.74	43.03	10.53	18.27	81.73	36.4
MW326766.1	29.4	13.36	44.89	12.34	25.71	74.29	29.95	23.58	31.6	10.14	44.81	13.44	23.58	76.42	45.4
MEAN	41.0	11.73	32.37	14.8	26.57	73.42	32.04	27.73	39.83	9.81	40.2	10.1	19.93	80	39.47
STD	5.17	1.74	5.18	0.84	1.65	1.65	1.72	3.82	5.37	2.12	5.19	1.96	2.33	2.33	2.89

ENC, effective number of codons; GC1, G + C content at the first position of codons; GC2, G + C content at the second position of codons; GC3, G + C content at the third positions of codons; AU3, A + U content at the third positions of codons.

Nucleotide content analysis at the 1st, 2nd, and 3rd synonymous codon positions disclosed that the values of GC1 ranged from 28.29 to 41.64% (mean: 32.04%; SD: 1.72), while the values of GC2 ranged from 23.38 to 46.77% (mean: 27.73%; SD: 3.82). Whereas, the values of GC3 ranged from 16.28 to 51.64% (mean: 19.93%; SD: 2.33), which are similar to previous studies on the Crimean–Congo hemorrhagic fever (CCHF) virus (38). In contrast, the values of AU3 ranged from 48.36 to 83.72% (mean: 78.11%; SD: 6.36) (Figure 1D). These data further supported the notion that an extensive area of LSDV is self-possessed of A/T contents (Table 1). This study supports the previous studies on the Alongshan virus and Zika virus (24, 39), while it seems to be inconsistent with a recent study on the hepatitis E virus (HEV) (40).

### Defining codon usage patterns

To describe and define the codon usage bias of LSDV, an RSCU analysis was carried out to determine why A/U nucleotides were favored at the third codon position. For 53 LSDV strains, the RSCU values of all synonymous codons were determined and compared to those of their hosts. The result showed that all the 18 most abundantly used codons in LSDV [TTT (Phe), TTA (Leu), ATT (Ile), GTT (Val), AGT (Ser), CCT (Pro), ACT (Thr), GCT (Ala), CAT (His), TAT (Tyr), CAA (Gln), AAT (Asn), AAA (Lys), GAT (Asp), TGT (Cys), CGT (Arg), and GGT (Gly)] ended with T or A (T: 14; A: 3). Interestingly, none of the preferred codons was G/C-ended. Thus, the A or T-end codon bases are shared more in the genome of LSDV, which is similar to an earlier research that T/A-ended codons have a high amount in the virus genome, such as chikungunya virus and Crimean–Congo hemorrhagic fever virus (34, 38). Furthermore, from the RSCU analysis, we found that the overrepresented (>1.6) codons are rarely seen in the genome of LSDV. Nearly all the ideal and non-ideal codons are located in the range of 0.6–1.6. We observed that most codons ending in A/T were overrepresented (>1.6), while codons ending in G/C were under-represented (<0.6) (Table 2), revealing that mutational pressure was the primary factor influencing codon usage patterns in LSDV, which was consistent with those given in previous studies (41, 42). From both the nucleotide content and RSCU analysis, we assumed that the selection of the preferred codons is generally inclined by compositional restraints, which determine the existence of mutational pressure. We are unsure of the fact that the compositional pressure could not be the single aspect related to LSDV patterns of codon usage, as although the total values of RSCU could disclose the pattern of codon usage for the genomes, it may conceal the codon usage variation among distinct genes in a genome (43).

**Table 2:** The relative synonymous codon usage frequency of Lumpy skin disease virus (LSDV) and its natural hosts (*Homo sapiens* and *Bos taurus*).

**AA**	**Codon**	**LSDV**	***Homo sapiens***	***Bos taurus***	**AA**	**Codon**	**LSDV**	***Homo sapiens***	***Bos taurus***
Phe	UUU	1.75	0.97	0.87	His	CAU	1.24	0.85	0.88
	UUC	0.24	1.03	1.13		CAC	0.75	1.15	1.12
Leu	UUA	3.36	0.50	1.71	Gln	CAA	1.89	0.49	0.71
	UUG	0.74	1.00	1.35		CAG	0.1	1.51	1.29
	CUU	1.06	0.81	0.73	Asn	AAU	1.54	0.98	0.87
	CUC	0.42	1.07	0.93		AAC	0.45	1.02	1.13
	CUA	0.32	0.46	0.58	Lys	AAA	1.53	0.88	0.89
	CUG	0.07	2.33	1.69		AAG	0.46	1.12	1.11
Ile	AUU	1.1	1.13	0.92	Asp	GAU	1.58	0.99	0.85
	AUC	0.13	1.37	1.01		GAC	0.41	1.01	1.15
	AUA	1.75	0.50	1.07	Glu	GAA	1.76	0.85	0.92
Val	GUU	1.89	0.79	0.69		GAG	0.23	1.15	1.08
	GUC	0.36	0.90	0.82	Cys	UGU	1.65	0.95	0.78
	GUA	1.55	0.52	0.72		UGC	0.34	1.05	1.22
	GUG	0.19	1.79	1.76	Arg	CGU	1.01	0.54	0.26
Ser	UCU	1.14	1.15	0.95		CGC	0.3	1.11	0.52
	UCC	0.45	1.17	1.06		CGA	0.32	0.76	0.27
	UCA	1.96	0.93	1.40		CGG	0.02	1.31	0.73
	UCG	0.58	0.36	0.43		AGA	4.14	1.18	2.16
	AGU	1.56	0.98	0.80		AGG	0.19	1.01	2.07
	AGC	0.29	1.42	1.53	Gly	GGU	1.43	0.71	1.51
Pro	CCU	1.06	1.20	0.94		GGC	0.02	1.35	1.01
	CCC	0.32	1.22	1.01		GGA	2.38	1.01	1.25
	CCA	2.31	1.14	1.45		GGG	0.15	0.93	1.23
	CCG	0.29	0.45	0.59					
Thr	ACU	1.02	1.03	0.87					
	ACC	0.44	1.32	1.09					
	ACA	1.07	1.19	1.44					
	ACG	0.45	0.46	0.60					
Ala	GCU	1.53	1.01	0.97					
	GCC	0.27	1.12	1.13					
	GCA	1.97	1.18	1.30					
	GCG	0.21	0.07	0.60					
Tyr	UAU	1.68	0.71	0.90					
	UAC	0.31	1.29	1.10					

AA, amino acid; the “RSCU” value represents the pattern of relative synonymous codon usage; over-represented (RSCU > 1.6), and under-represented (RSCU <0.6) codons are marked with Red and green, respectively; and the favored codons for Lumpy skin disease virus are underlined.

**Figure 2:**
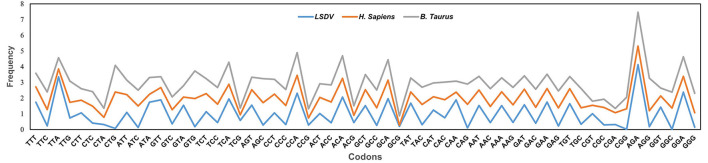
Comparative analysis of relative synonymous codon usage (RSCU) patterns between lumpy skin disease virus (LSDV), and its hosts *Bos taurus* and *Homo sapiens*. X-axis represents the Codons, while the Y-axis represents the Frequency.

Additionally, to determine whether the codon usage bias of LSDV can be constrained by its hosts (*Bos taurus* and *Homo sapiens*), all their codon RSCU values were also calculated (Table 2). This study indicated that 12 of 59 synonymous codons of LSDV are equivalent to those of *Homo sapiens*, individually, and that 09 of 59 synonymous codons are equivalent to those of *Bos taurus* (Figure 2, Table 2). Here, the role of selection from the *Bos taurus* in shaping codon usage patterns of LSDV is different from that of the host *Homo sapiens*. It was suggested that the similarity of codon usage patterns between LSDV and *Bos taurus*/*Homo sapiens* enhances the efficiency of translation in the virus genome (44).

### Use of codon biases in lumpy skin disease virus

To determined the magnitude of CUB within LSDV coding sequences, the gene's ENC value was assessed and plotted next to the GC content at the 3rd codon position (GC3) (Table 1). Here, the values of ENC were shown to vary from 36.28 to 48.18, indicating a high level of genetic difference in the codon's usage. Nevertheless, the average value of ENC was 39.47 > 20, implying that the whole CUB was low (Table 1), which was also observed in Human cytomegalovirus (45), Ebola virus (46), and Porcine astrovirus (18). The ENC analysis revealed that a low codon bias was seen along with the position of natural selection on the genes (32, 47). Therefore, it seems that the evolution of a low codon bias within LSDV coding sequences has enabled LSDV survival within the host, even though the host possesses codon usage preferences distinct from those of LSDV.

**Figure 3:**
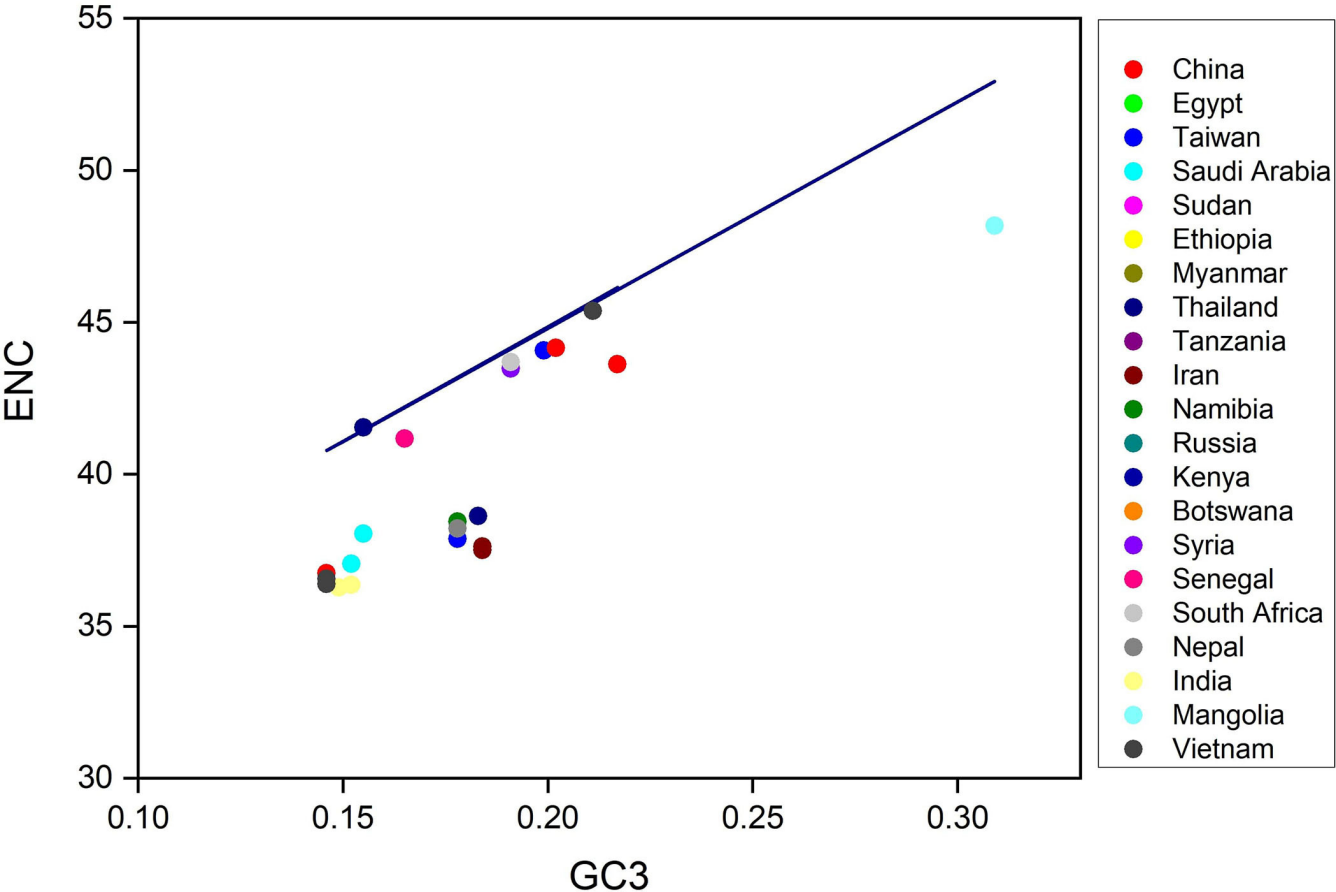
ENC-GC3 plots of 53 lumpy skin disease virus (LSDV) strains: The effective number of codons (ENC-values, Y-axis) is plotted against the GC content at the third synonymous codon positions (GC3-values, X-axis).

Next, to determine the codon usage of the genes, a distribution plot that deviated from a similar usage of indistinguishable codons was employed. Here, ENC values were used against the GC3s. If the GC subject of the gene exhibits mutational pressure, all the points in this plot lie below or close to the expected curve, indicating a random codon usage. However, if there is selection pressure on the gene, all the points lie on or below the expected curve. Here, we plotted the ENC values of each gene against the GC3 content (Figure 3). The results reveal that mutational pressure and natural selection both influence the codon usage pattern of an LSDV genome, as the majority of the points fall below the expected curve and just a few points beyond it (48, 49).

### Neutrality plot analysis

**Figure 4:**
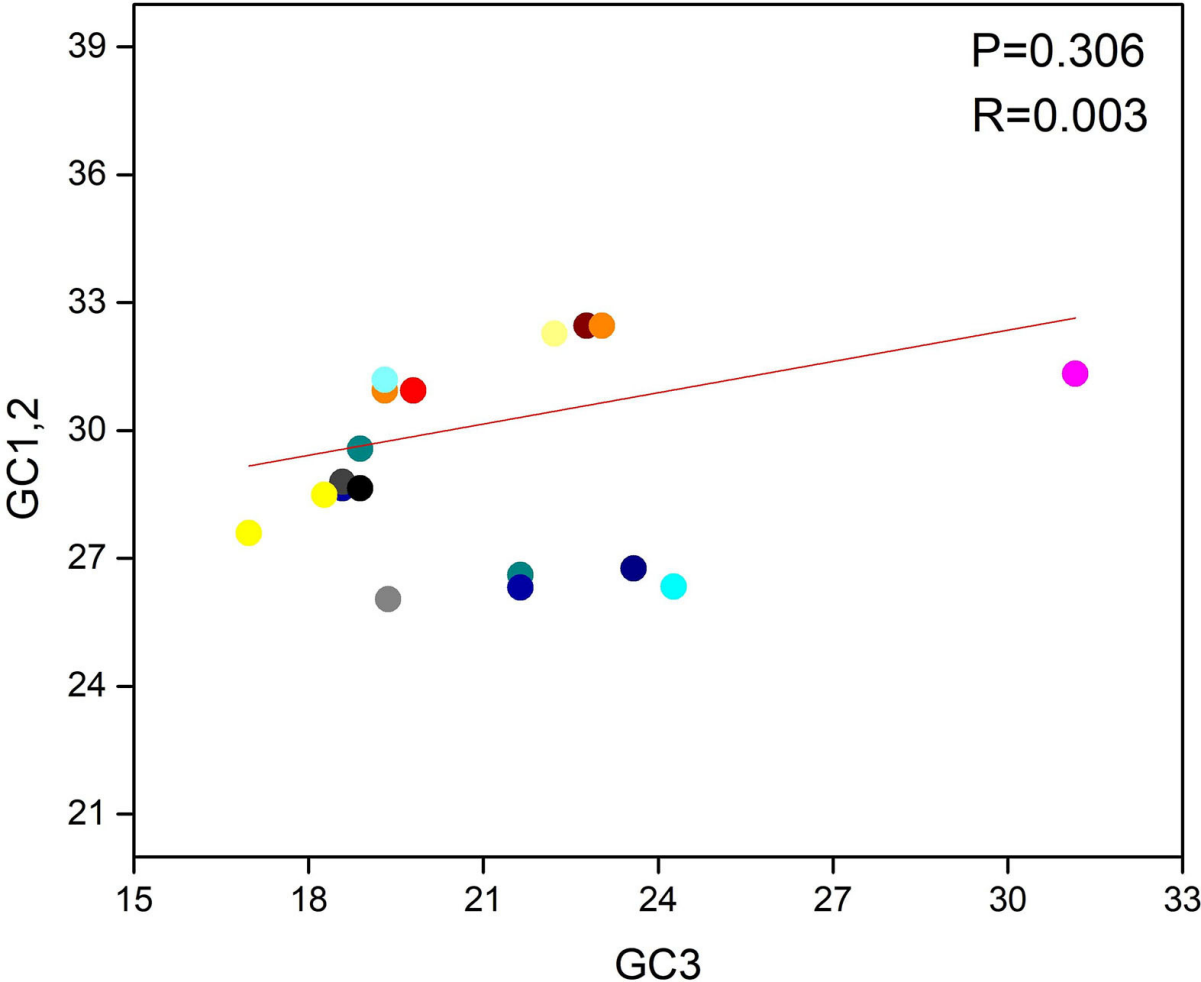
Neutrality plot between (GC3 vs. GC1, 2) for the entire coding sequence of lumpy skin disease virus (LSDV). GC1, 2 represent GC at 1st and 2nd codon positions. While GC3 represents GC at 3rd codon position. And the orange solid line represents the regression analysis of GC1, 2 against GC3.

A neutrality plot analysis was performed, which implied the bond between GC1/2 and GC3 composition, to determine the position of mutation and selection pressure that has an impact on the form of the codon usage bias (CUB). To observe the association, we programmed a paradigm on the plot of neutrality between GC3 and GC1/2 for the LSDV genome. Here, the said plot shows that no significant relation was found between GC3 and GC1/2 contents because the regression value and link are *P* > 0.05 and *r* = 0.003 (Figure 4). Finally, we suggested that both natural selection and mutational pressure have an impact on the codon usage shaping of LSDV. This phenomenon is similar to those given in the previous studies (41, 50, 51).

### Adaptation of lumpy skin disease virus to the host genome

The codon adaptation index (CAI) analysis was performed to regulate the optimization of codon usage and LSDV adaptation to its hosts (52). The values of CAI range from 0 to 1; a value near 1 indicates higher levels of codon usage bias (13). For all codons, the CAI values were measured through the reference of *Bos taurus* and *Homo sapiens* codon usage. We determined that, as regards *Bos taurus* and *Homo sapiens*, the mean CAI values of LSDV coding regions were 0.59 and 0.68 (>0.5), which revealed that LSDV has a good adaptation to its hosts and a minimal translation pressure (Supplementary Figure 1) (24, 53). The tendency of *Homo sapiens* to move toward a high CAI value recommends that the selection pressure from *Homo sapiens* should impact the LSDV codon usage, and that the codon usage evolution in LSDV should permit it to use the translation machinery of *Homo sapiens* more capably. Our result was consistent with the published work (54).

To check if the observed statistically significant differences arose in the values of CAI (29, 38), the values of expected CAI (e-CAI) were considered for LSDV CDS with *Bos taurus* and *Homo sapiens* codon usage sets. The results of the e-CAI values were 0.70 and 0.79 (*P* < 0.05) in relation to *Bos taurus* and *Homo sapiens*, revealing that the generated sequences keep to a normal distribution. The outcomes of this study about the preferences of codon usage are comparable with those of previous research (12, 55).

### Discrepancy in the usage of codon in lumpy skin disease virus

**Figure 5:**
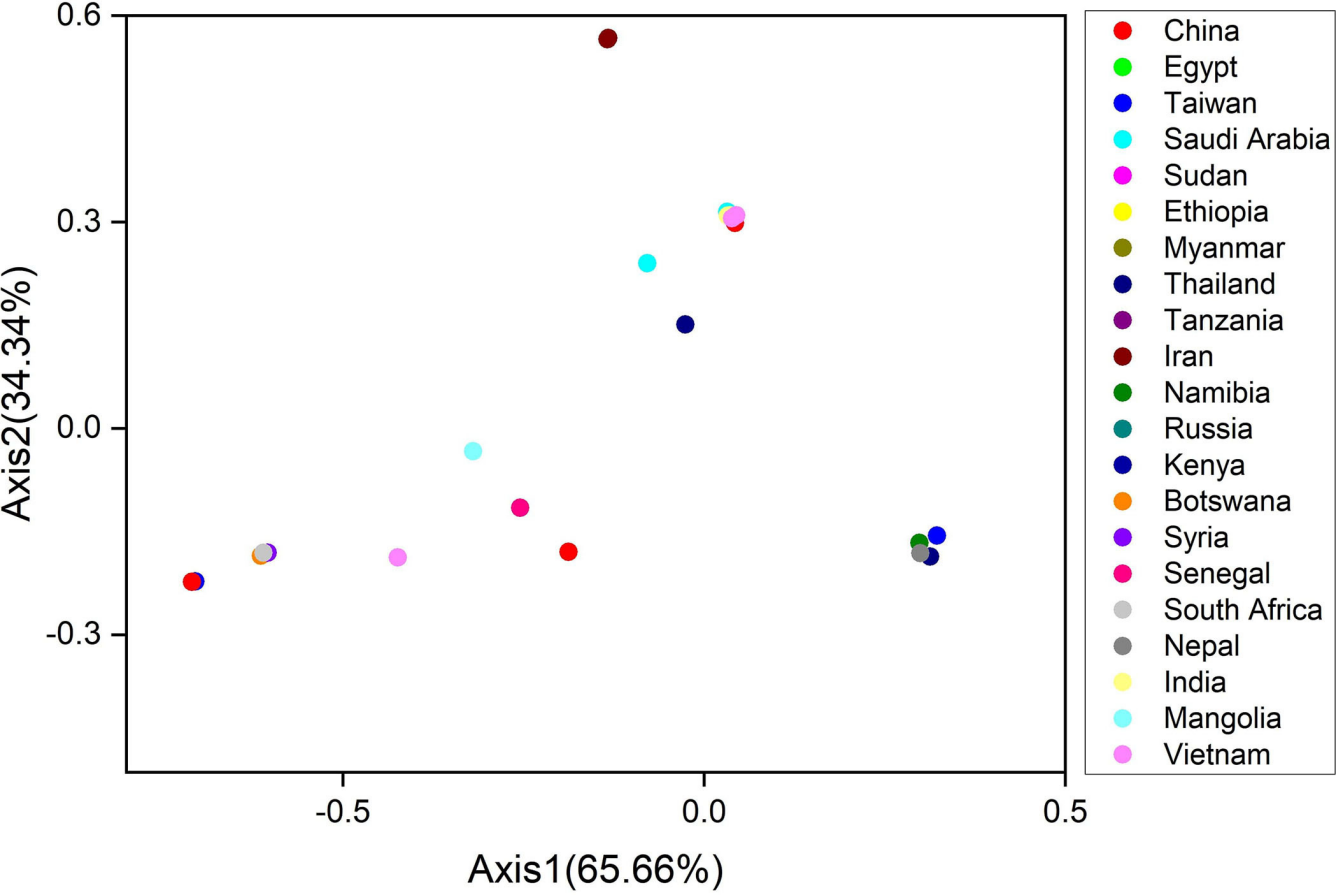
The correspondence analysis (COA) of the genes in lumpy skin disease virus (LSDV) genomes. Each point represents a gene corresponding to the coordinates of the first and second axes of variation generated from the correspondence analysis.

The Correspondence (CO) analysis describes the discrepancy in the usage of codons. The changes that occur in the patterns of codon usage are revealed through RSCU values. In the plot of CO analysis, axes 1 and 2 are the two main factors of general discrepancy (30, 34, 56). We used the values of these two axes to draw COA plots, where each strain is represented by a point, and the distance between strains gives a degree of similarity or dissimilarity in the codon usage patterns. The principal axes 1 and 2 accounted for the total variation: 65.66 and 34.34% (Figure 5). These results propose the fact that axis 1 signifies the LSDV strains and axis 2 signifies the countries where the LSDV arises. Scattered data on the main axis represent various geographical ancestries and their relationships. All the LSDV strains were found to be placed in groups using COA (Figure 5). The entire Iran LSDV strains were assembled into one clade, while strains from Taiwan, Nepal, Namibia, and Thailand were present in an alternative clade. Moreover, China, Saudi Arabia, and Sudan strains were divided into separate groups (Figure 5). These studies reveal that the topographical sites play a major role in the evolution of LSDV and in a synonymous codon usage pattern, where in the future such investigations may assist in discovering the essence of rising LSDV strains. Furthermore, present outcomes also show that more than one widespread genetic lineage was found in every infected country.

**Figure 6:**
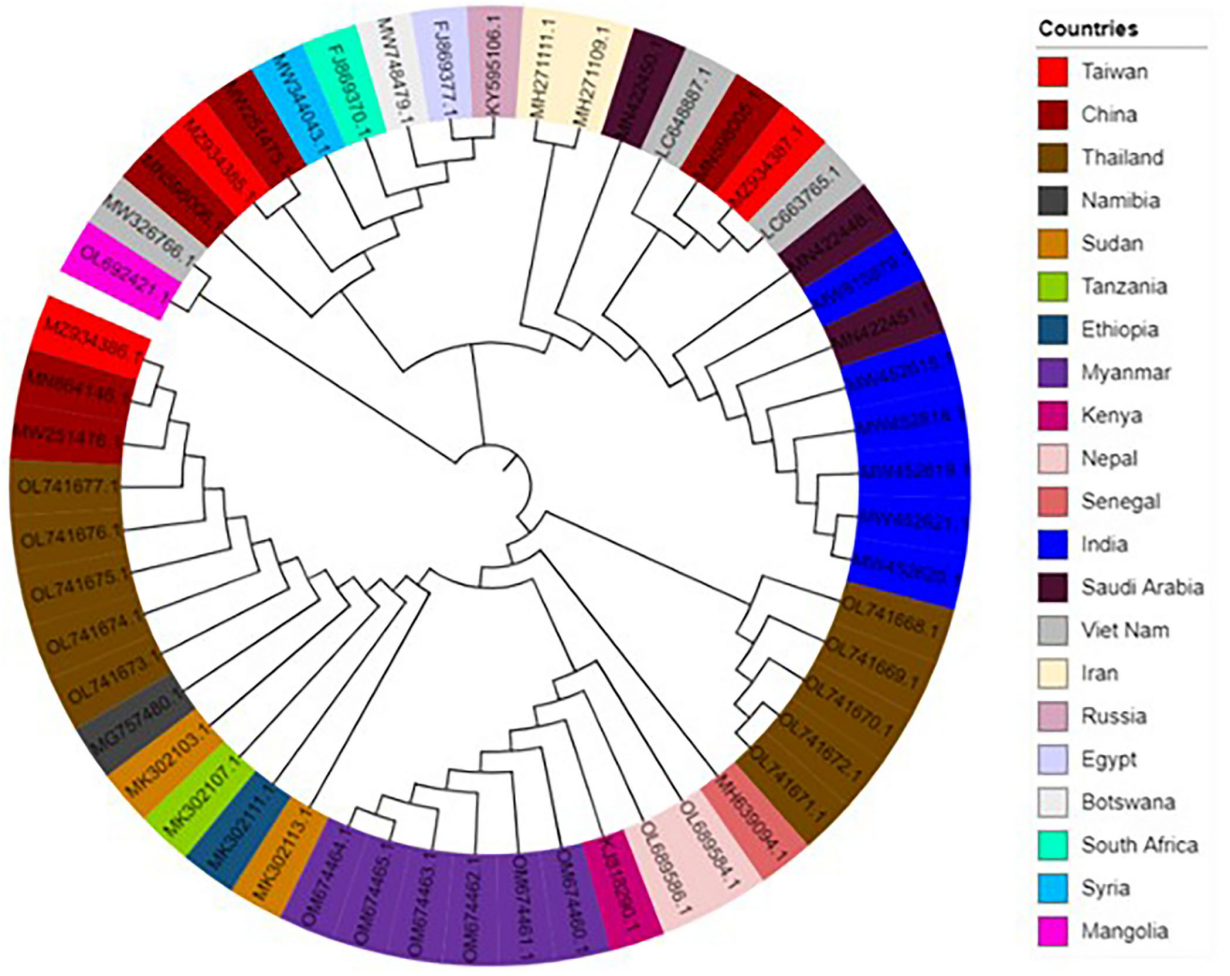
Phylogenetic tree based on the polyprotein-coding regions of 53 lumpy skin disease virus (LSDV) strains. The tree was generated by the maximum-likelihood (ML) method using Clustal X2. The tree was designed by using the online tool “iTOL”.

To assess the consequence of evolutionary procedures in the LSDV codon usage pattern, a phylogenetic analysis was carried out using the maximum-likelihood (ML) method. The entire LSDV that separates is dispersed throughout the world, as evidenced by the phylogenetic tree, which shows that no strains form a cluster among different individual countries (Figure 6). The study suggested that this virus might have become altered due to some specific geographical effects such as climatic changes and environmental changes, which support the main outcome of evolutionary processes and topographical dispersal on codon usage patterns. The current study further exposed the signs of recombination and genome reassortment during single host co-infection, signifying the potential for the upcoming arrival of the novel alternates (57, 58).

### Codon usage pattern dominating effects on lumpy skin disease virus

**Figure 7:**
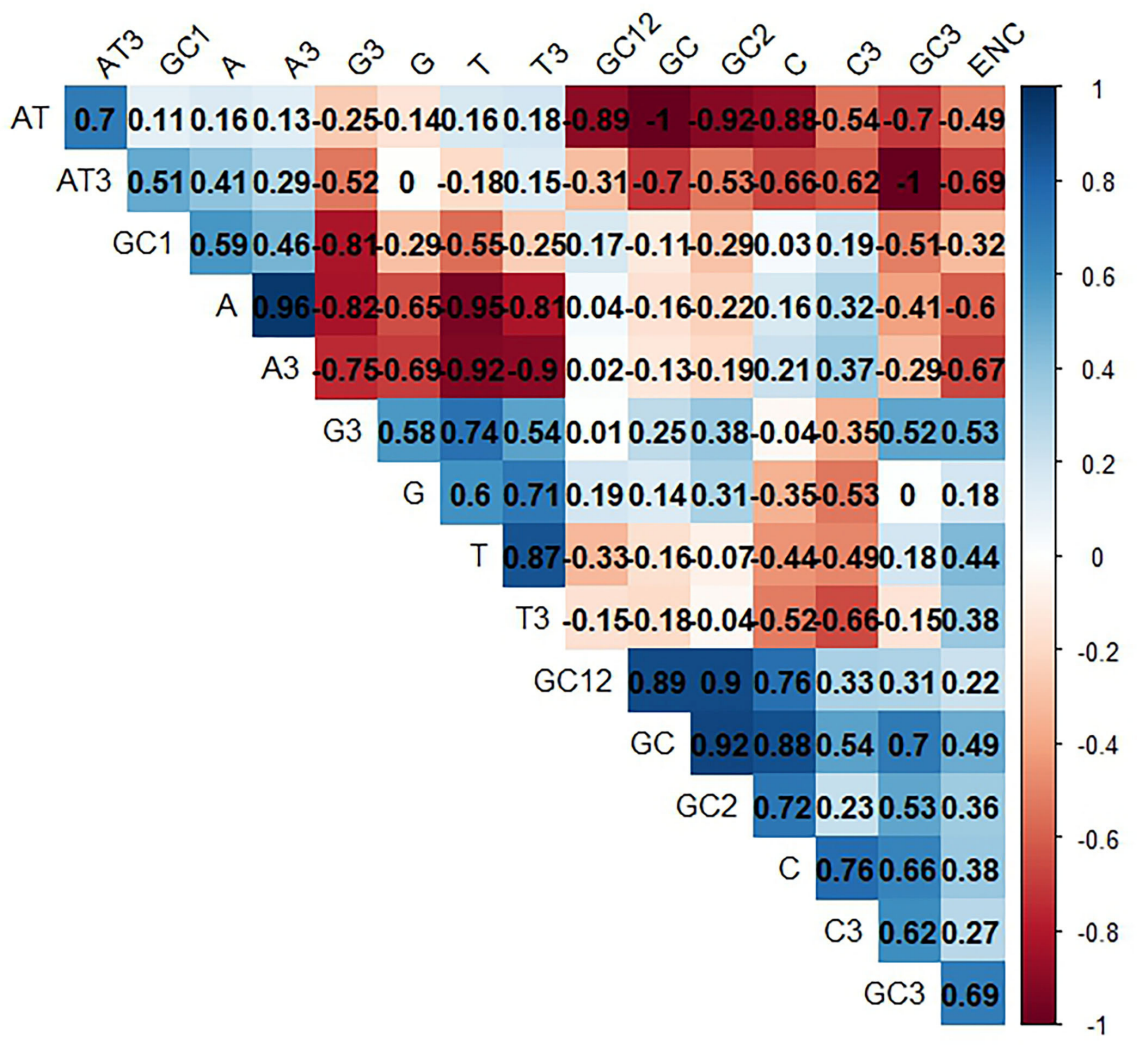
Correlation analysis among different nucleotide contents of lumpy skin disease virus (LSDV). The dark blue means positive correlation, and the dark red means negative correlation; the larger the value means more significant correlation.

Here, we consider two factors: natural selection and mutational pressure, to determine the codon usage bias (CUB) in LSDV. Accordingly, we performed a correlation analysis between total nucleotide contents (A, G, C, T), nucleotide contents at 3rd codon position, GC contents (1st, 2nd, 3rd), GC12, and ENC. The ENC values of the LSDV sequences seemed to show a positive relationship with T, C, G, T3, C3, G3, GC, GC2, GC3, and GC12, except A, A3, AT, GC1, and AT3, which have a negative relationship that probably affects the LSDV codon usage pattern (Figure 7). Previous studies have suggested that when we have the base compositions at the 3rd position of codon, mutational bias is mostly explained, while with base compositions at the 1st and 2nd positions, selective pressure is mostly validated (45, 59).

Such an impact was also observed among A, G, T, C, GC, AT, GC3, AT3, A3, C3, G3, and T3 with GC12. The A, G, C, G3, GC, GC3, A3, and C3 have a positive correlation with ENC, while the T, AT, AT3, and T3 have a negative correlation with ENC. This result implies the significance of mutational and selection pressure in getting the LSDV codon usage pattern (Figure 7). Additionally, it also suggests that the contents of a nucleotide have an impact on the codon usage pattern of LSDV (60).

### Similarity index analysis

**Figure 8:**
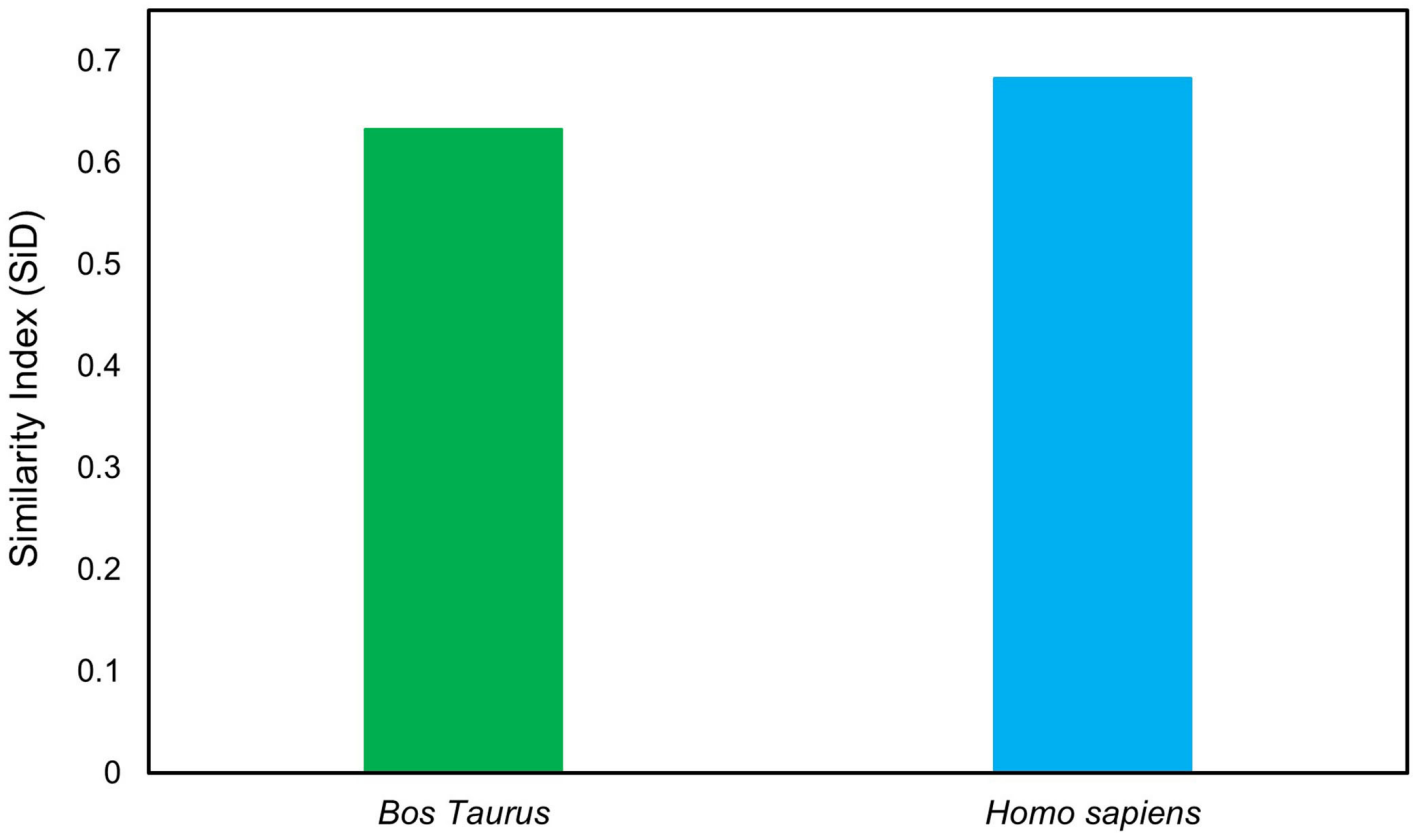
Similarity index (SiD) analysis of the codon usage between lumpy skin disease virus (LSDV) and its hosts.

The similarity index (SiD) analysis was carried out to assess the potential impact of *Bos taurus* and *Homo sapiens* codon usage patterns on the evolution of the codon usage patterns of the LSDV. The SiD was found to be nearly similar in both *Bos taurus* (0.63) and *Homo sapiens* (0.68), indicating that both of them have a dominant influence on the formation of LSDV codon usage patterns (Figure 8). Previous studies showed that *Bos taurus* is thought to be the principal reservoir and a host of LSDV. It is likely that the virus has stabilized its genetic traits to better adapt to the environment of its primary host (35, 61).

## Data availability statement

Publicly available datasets were analyzed in this study. The names of the repository/repositories and accession number(s) can be found in the article/Supplementary material.

## Author contributions

SRah and IR: conceptualization, methodology, software, data curation, and writing—original draft preparation. AR, SRaz, and NH: helped in write-up and editing and validation. HR: methodology, visualization, and validation. AA, MA, and HS: reviewing and editing and validation. All authors contributed to the article and approved the submitted version.

## Conflict of interest

The authors declare that the research was conducted in the absence of any commercial or financial relationships that could be construed as a potential conflict of interest.

## Publisher's note

All claims expressed in this article are solely those of the authors and do not necessarily represent those of their affiliated organizations, or those of the publisher, the editors and the reviewers. Any product that may be evaluated in this article, or claim that may be made by its manufacturer, is not guaranteed or endorsed by the publisher.

## Supplementary material

Supplementary Figure 1 Codon adaptation index (CAI) of lumpy skin disease virus (LSDV) to its hosts (*Bos taurus* and *Homo sapiens*).

Supplementary Table 1 Detailed information of lumpy skin disease virus studied in this study.
