# Supplementary material for: Analysis of codon usage bias of lumpy skin disease virus causing livestock infection
Source: Front Vet Sci. 2022 Dec 5;9:1071097. doi: 10.3389/fvets.2022.1071097 (PMC9762553; doi:10.3389/fvets.2022.1071097)
Supplement: Supplementary Figure 1 — Codon adaptation index (CAI) of lumpy skin disease virus (LSDV) to its hosts (Bos taurus and Homo sapiens). [file Data_Sheet_1.zip › Table 1.DOCX]

**Table S1:** Detailed information of lumpy skin disease virus studied in this study.

| S. No | Accession number | Country | Sequence type | Length (bp) |
| --- | --- | --- | --- | --- |
| 1 | MN864146.1 | China | Complete CDS | 1236 |
| 2 | FJ869377.1 | China | Complete CDS | 1134 |
| 3 | MW251475.1 | China | Complete CDS | 1146 |
| 4 | FJ869377.1 | Egypt | Complete CDS | 1134 |
| 5 | MZ934387.1 | Taiwan | Complete CDS | 969 |
| 6 | MZ934386.1 | Taiwan | Complete CDS | 606 |
| 7 | MZ934385.1 | Taiwan | Complete CDS | 1146 |
| 8 | MN422450.1 | Saudi Arabia | Complete CDS | 969 |
| 9 | MN422448.1 | Saudi Arabia | Complete CDS | 969 |
| 10 | MN422451.1 | Saudi Arabia | Complete CDS | 969 |
| 11 | MN598006.1 | China | Complete CDS | 1146 |
| 12 | MN598005.1 | China | Complete CDS | 969 |
| 13 | MK302113.1 | Sudan | Complete CDS | 606 |
| 14 | MK302103.1 | Sudan | Complete CDS | 606 |
| 15 | MK302111.1 | Ethiopia | Complete CDS | 606 |
| 16 | OM674465.1 | Myanmar | Complete CDS | 606 |
| 17 | OM674464.1 | Myanmar | Complete CDS | 606 |
| 18 | OM674463.1 | Myanmar | Complete CDS | 606 |
| 19 | OM674462.1 | Myanmar | Complete CDS | 606 |
| 20 | OM674461.1 | Myanmar | Complete CDS | 606 |
| 21 | OM674460.1 | Myanmar | Complete CDS | 606 |
| 22 | OL741677.1 | Thailand | Complete CDS | 606 |
| 23 | OL741676.1 | Thailand | Complete CDS | 606 |
| 24 | OL741675.1 | Thailand | Complete CDS | 606 |
| 25 | OL741674.1 | Thailand | Complete CDS | 606 |
| 26 | OL741673.1 | Thailand | Complete CDS | 606 |
| 27 | OL741672.1 | Thailand | Complete CDS | 636 |
| 28 | OL741671.1 | Thailand | Complete CDS | 636 |
| 29 | OL741670.1 | Thailand | Complete CDS | 636 |
| 30 | OL741669.1 | Thailand | Complete CDS | 636 |
| 31 | OL741668.1 | Thailand | Complete CDS | 636 |
| 32 | MK302107.1 | Tanzania | Complete CDS | 606 |
| 33 | MH271111.1 | Iran | Complete CDS | 513 |
| 34 | MH271109.1 | Iran | Complete CDS | 513 |
| 35 | MG757480.1 | Namibia | Complete CDS | 606 |
| 36 | KY595106.1 | Russia | Complete CDS | 1134 |
| 37 | KJ818290.1 | Kenya | Complete CDS | 606 |
| 38 | MW748479.1 | Botswana | Complete CDS | 1134 |
| 39 | MW344043.1 | Syria | Complete CDS | 1134 |
| 40 | MH639094.1 | Senegal | Complete CDS | 1146 |
| 41 | FJ869370.1 | South Africa | Complete CDS | 1134 |
| 42 | OL689586.1 | Nepal | Complete CDS | 606 |
| 43 | OL689584.1 | Nepal | Complete CDS | 606 |
| 44 | MW815879.1 | India | Complete CDS | 969 |
| 45 | MW452621.1 | India | Complete CDS | 969 |
| 46 | MW452620.1 | India | Complete CDS | 969 |
| 47 | MW452619.1 | India | Complete CDS | 969 |
| 48 | MW452618.1 | India | Complete CDS | 969 |
| 49 | MW452615.1 | India | Complete CDS | 969 |
| 50 | OL692421.1 | Magnolia | Complete CDS | 876 |
| 51 | LC648887.1 | Viet Nam | Complete CDS | 969 |
| 52 | LC663765.1 | Viet Nam | Complete CDS | 969 |
| 53 | MW326766.1 | Viet Nam | Complete CDS | 1272 |
