# Supplementary figures and images for: Analysis of codon usage bias of lumpy skin disease virus causing livestock infection
Source: Front Vet Sci. 2022 Dec 5;9:1071097. doi: 10.3389/fvets.2022.1071097 (PMC9762553; doi:10.3389/fvets.2022.1071097)

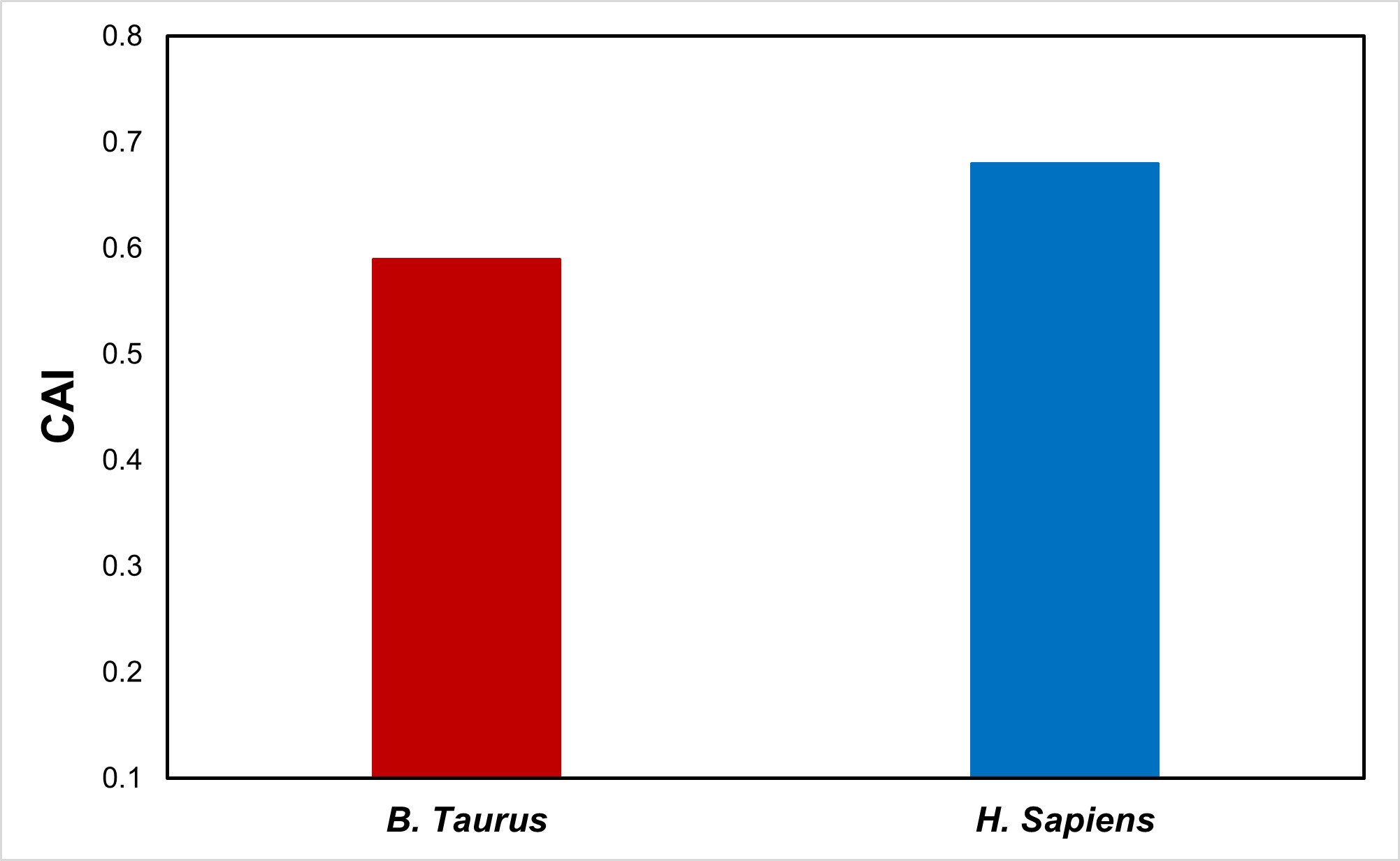

Supplement: Supplementary Figure 1 — Codon adaptation index (CAI) of lumpy skin disease virus (LSDV) to its hosts (Bos taurus and Homo sapiens). [file Data_Sheet_1.zip › Image 1.JPEG]
